# Association between *GRN* rs5848 Polymorphism and Parkinson′s Disease in Taiwanese Population

**DOI:** 10.1371/journal.pone.0054448

**Published:** 2013-01-16

**Authors:** Kuo-Hsuan Chang, Chiung-Mei Chen, Yi-Chun Chen, Ya-Chin Hsiao, Chin-Chang Huang, Hung-Chou Kuo, Hsuan-Chu Hsu, Guey-Jen Lee-Chen, Yih-Ru Wu

**Affiliations:** 1 Department of Neurology, Chang Gung Memorial Hospital, Chang Gung University College of Medicine, Taipei, Taiwan; 2 Department of Life Science, National Taiwan Normal University, Taipei, Taiwan; Centre Hospitalier Universitaire Vaudois (CHUV), Switzerland

## Abstract

A single nucleotide polymorphism GRN rs5848 (3′UTR+78 C>T) was reported to alter the risk for frontotemporal lobar degeneration. Herein, we investigated the effect of *GRN* rs5848 on the risk of Parkinson’s disease (PD) by genotyping 573 Taiwanese patients with PD and 490 age-matched control subjects. Compared to subjects with CC genotype, those with TT genotype had a 1.58-fold increased risk of PD (95% CI: 1.77∼2.34, *P* = 0.021). PD patients demonstrate a higher frequency of T allele (37.2%) than controls (32.2%; odds ratio [OR] = 1.24, 95% CI: 1.04∼1.49, *P* = 0.017). This susceptibility was particularly observed in female subjects, in which TT genotype had a 2.16-fold increased risk of PD as compared with controls(95% CI: 1.24∼3.78, *P* = 0.006). The frequency of T allele (39.3%) in female PD patients was higher than in female control subjects (31.1%; OR = 1.43, CI: 1.11∼1.87, *P* = 0.007). No association was observed between *GRN* rs5848 and susceptibility in male subjects. These findings show that the *GRN* rs5848 TT genotype and T allele are risk factors for female Taiwanese patients with PD.

## Introduction

Parkinson’s disease (PD) is the second most common neurodegenerative disorder worldwide [1]. It affects 1% of the population aged over sixty, and is characterized by a slowness of movement and a difficulty in initiating movement [1]. The pathogenesis of PD includes progressive degeneration of the dopaminergic neurons and the presence of Lewy bodies with enrichment of α-synuclein in the ventral midbrain [2]. This cell death results in a deficiency of dopamine in the striatum. When the level of striatal dopamine falls below 70∼80%, the clinical presentations of PD are manifested [1].

The *GRN* gene (granulin, MIM#138945) encodes an 88-kDa secreted growth factor progranulin [3], which is involved in multiple physiological functions, including wound healing, tumor growth, and embryonic brain development [4], [5], [6]. A single nucleotide polymorphism (SNP) in the 3′-untranslated region of *GRN* (3′UTR+78C>T; rs5848) was reported to alter the risk for frontotemporal lobar degeneration (FTLD) [7]. Although *GRN* rs5848 polymorphism was not associated with the risk of PD in Caucasian populations [8], its effects in other ethnic genetic and environmental backgrounds is unknown. Therefore, we assessed whether *GRN* rs5848 polymorphism contributes to the genetic etiology of PD by using case-control analysis in 573 Taiwanese patients with PD and 490 control subjects.

## Results

In total, 1063 subjects, including 573 patients (female/male: 253/320) with PD and 490 normal controls (female/male: 249/241, *P* = 0.030), were recruited. Only 1 proband with familial PD in the same family was included to minimize the skew caused by the other family members carrying the same genetic polymorphism. *GRN* rs5848 TT genotype showed a higher prevalence in PD than CC genotype did (odds ratio [OR] = 1.63, 95% CI: 1.10 ∼ 2.42, *P* = 0.015, Table 1). This finding was also present in the recessive model on the borderline of statistical significance (OR = 1.51, 95% CI: 1.04 ∼ 2.19, *P* = 0.031). The T allele showed a greater frequency in PD than the C allele did (OR = 1.25, 95% CI: 1.05 ∼ 1.50, *P* = 0.014). These findings were absent in Caucasian populations [8]. The distributions of the genotypes and the minor allele frequency did not differ between Taiwanese and Caucasians in the control group.

**Table 1:** Frequency of genotype and allele polymorphisms of *GRN* rs5848 among Parkinson’s disease (PD) patients and controls in Taiwanese and Caucasian.

	PD (%)	Controls (%)	OR (95% CI)	*P* value
**Taiwan**				
Genotype frequency				
CC	231 (40.3%)	226 (46.1%)	1.00	
CT	258 (45.0%)	212 (43.3%)	1.17 (0.91 ∼ 1.52)	0.227#
TT	84 (14.7%)	52 (10.6%)	1.63 (1.10 ∼ 2.42)	0.015#
Dominant model				
CC	231 (40.3%)	226 (46.1%)	1.00	
CT+TT	342 (59.7%)	264 (53.9%)	1.26 (0.99 ∼ 1.62)	0.062#
Recessive model				
CT+CC	489 (85.7%)	438 (89.4%)	1.00	
TT	84 (14.7%)	52 (10.6%)	1.51 (1.04 ∼ 2.19)	0.031#
Allele frequency				
Major allele (C)	720 (62.8%)	664 (67.8%)	1.00	
Minor allele (T)	426 (37.2%)	316 (32.2%)	1.25 (1.05 ∼ 1.50)	0.014#
**Caucasian** *				
Genotype frequency				
CC	361 (46.8%)	312 (48.6%)	1.00	
CT	324 (42.0%)	263 (41.0%)	1.07 (0.85 ∼ 1.33)	0.580‡
TT	86 (11.2%)	67 (10.4%)	1.11 (0.78 ∼ 1.58)	0.565‡
Dominant model				
CC	361 (46.8%)	312 (48.6%)	1.00	
CT+TT	410 (53.2%)	330 (51.4%)	1.07 (0.84 ∼ 1.32)	0.506‡
Recessive model				
CT+CC	685 (88.8%)	575 (89.4%)	1.00	
TT	86 (11.2%)	67 (10.4%)	1.08 (0.77 ∼ 1.51)	0.665‡
Allele frequency				
Major allele (C)	1046 (67.8%)	887 (69.1%)	1.00	
Minor allele (T)	496 (32.2%)	397 (30.9%)	1.06 (0.90 ∼ 1.24)	0.478‡

OR: odds ratio.

* Genotype and allele frequencies of *GRN* rs5848 are from Jasinska-Myga et al., 2009 [8].

# *P* value of binary logistic regression with adjustment of age and gender.

‡ *P* values of Chi square test.

The unequal gender distribution between PD and control groups may influence the prevalence between the *GRN* rs5848 genotype/allele and PD. Thus, we stratified our groups according to gender (Table 2). In female subjects, the TT genotype showed a significantly greater frequency in PD than did the CC genotype (OR = 2.99, 95% CI: 1.50 ∼ 5.95, *P* = 0.002). The recessive model represented this high frequency between TT genotype and PD (OR = 2.85, 95% CI: 1.48 ∼ 5.48, *P* = 0.002). The occurrence of the T allele in female PD patients was higher than that in female control subjects (OR = 1.59, 95% CI: 1.16 ∼ 2.18, *P* = 0.004). The distributions of the genotypes and the minor allele frequency did not differ between PD and controls in male subjects. Both female and male control subjects displayed similar distributions of genotypes and the minor allele.

**Table 2:** Frequency of genotype and allele polymorphisms of *GRN* rs5848 among Parkinson’s disease (PD) patients and controls in female and male patients.

	PD (%)	Controls (%)	OR (95% CI)	*P* value
**Female**				
Genotype frequency				
CC	99 (39.1%)	119 (47.8%)	1.00	
CT	109 (43.1%)	105 (42.2%)	1.11 (0.71 ∼ 1.76)	0.643
TT	45 (17.8%)	25 (10.0%)	2.99 (1.50 ∼ 5.95)	0.002
Dominant model				
CC	99 (39.1%)	119 (47.8%)	1.00	
CT+TT	154 (60.9%)	130 (52.2%)	1.42 (0.93 ∼ 2.17)	0.103
Recessive model				
CT+CC	208 (82.2%)	224 (90.0%)	1.00	
TT	45 (17.8%)	25 (10.0%)	2.85 (1.48 ∼ 5.48)	0.002
Allele frequency				
Major allele (C)	307 (60.7%)	343 (68.9%)	1.00	
Minor allele (T)	199 (39.3%)	155 (31.1%)	1.59 (1.16 ∼ 2.18)	0.004
**Male**				
Genotype frequency				
CC	132 (41.3%)	107 (44.4%)	1.00	
CT	149 (46.5%)	107 (44.4%)	1.24 (0.86 ∼ 1.20)	0.251
TT	39 (12.2%)	27 (11.2%)	1.14 (0.64 ∼ 2.03)	0.649
Dominant model				
CC	132 (41.3%)	107 (44.4%)	1.00	
CT+TT	188 (58.7%)	134 (55.6%)	1.22 (0.86 ∼ 1.74)	0.265
Recessive model				
CT+CC	281 (87.8%)	214 (88.80%)	1.00	
TT	39 (12.2%)	27 (11.2%)	1.02(0.59 ∼ 1.76)	0.941
Allele frequency				
Major allele (C)	413 (64.5%)	321 (66.6%)	1.00	
Minor allele (T)	227 (35.5%)	161 (33.4%)	1.12 (0.86 ∼ 1.45)	0.395

OR: odds ratio.

*P* value of logistic regression with adjustment of age.

## Discussion

The present study showed that the *GRN* rs5848 SNP affects the risk of developing PD in Taiwanese population. Our results differ from those of Jasinska–Myga et al. (2009), who reported a lack of association between PD and the *GRN* rs5848 T allele and TT genotype in patients with PD in the US and Poland [9]. This discrepancy demonstrates the differential effect of *GRN* rs5848 on PD risk between Eastern and Western populations.

A number of genetic variants exert population-specific influences on the risk of developing PD. For example, *LRRK2* G2835R and R1628P are common polymorphisms in Taiwan and Singapore [9], [10], [11]. By contrast, these associations were not observed in Caucasian populations [12], [13]. SNPs rs11931532, rs12645693, rs4698412, and rs4538475 of *BST1* are identified as risk factors for PD in a Japanese population [14], while this association has not been described in Chinese populations [15]. To further understand the role of *GRN* rs5848 in determining PD in ethnology, more genetic epidemiological studies should be performed in other races.

Several studies have shown that cerebral spinal fluid, serum, and plasma progranulin levels are significantly lower in *GRN* mutation carriers than in non-carriers [16], [17], [18]. Reduced levels of progranulin could affect both neuronal survival and CNS inflammatory processes [19], [20], which leads to loss of neurons. To date, more than 80 mutations of *GRN* have been found in neurodegenerative diseases [http://www.hgmd.cf.ac.uk/ac/gene.php?gene=GRN]. Similar to our results, patients homozygous for the *GRN* rs5848 T allele are more prone to developing FTLD than are homozygous C-allele carriers [7]. The *GRN* rs5848 SNP is predicted to create a micro-RNA 659 (miR-659) binding site [7]. Specifically, *GRN* rs5848 T increases the binding of miR-659 compared with the C allele, which thereafter increases translational suppression of *GRN* [7]. Reduced serum level of the progranulin was identified in homozygous *GRN* rs5848 T-allele carriers [21], supporting the hypothesis that *GRN* rs5848 affects the risk of neurodegenerative diseases by regulating *GRN* expression.

In elderly populations, men are approximately 2 times more likely to develop PD than are women [22], [23]. The factors contributing to the male prevalence of PD are unknown. Environmental factors, such as work-related exposure to toxins or heavy metals and pesticides, may be present at lower levels in the female living environment, which may result in the lower prevalence of PD in female populations [22]. Women may also benefit from putative protective factors, such as early life exposures to endogenous estrogen [24], [25], [26]. Further, there may also be gender-related differences in expression of genes related to signaling pathways associated with PD [27], [28]. Although the present study showed that the *GRN* rs5848 SNP modifies the risk of PD, particularly in the female populations of Taiwan, a large-scale study on female PD patients should be carried out to consolidate this gender-specific association.

An association between PD and *GRN* has not been described in published PD- genome-wide association studies (PD-GWAS) in North American, European, and Asian populations [14], [29], [30], [31], [32], [33], [34], [35], [36]. This is because PD is probably a multifactorial disorder for which a number of modest risk factors, over a lifespan, may contribute to ethnic or geographic differences of genetic susceptibility in PD patients. In support of this is the observation of an interaction between coffee and the glutamate receptor gene, *GRIN2A*, in PD [37]. Thus it is more likely that *GRN* rs5848 plays the role of a PD risk modifier by interaction with unknown environmental factors in Taiwan, which may explain the negative results in the available PD-GWAS.

Although our result is significant, there are limitations in this study. The single SNP analysis does not clarify the association between other regions around or within the gene and PD. The role of gene-gene or gene-environmental interaction has not been evaluated. In addition, the sample size in our study may not be able to identify an association when the genetic effect of the allele is less than 1.5. Nevertheless, our finding indicates the potential of *GRN* rs5848 T allele as a genetic risk factor in PD patients, particularly in the female population. More research into the influence of SNPs in *GRN* on PD onset or progression is clearly warranted.

## Subjects and Methods

### Ethics Statement

This study was performed according to a protocol approved by the institutional review boards of Chang Gung Memorial Hospital (ethical license No: 98-3980A3), and all examinations were performed after obtaining written informed consents.

### Patient Population

Patients diagnosed with PD were recruited from the neurology clinics of Chang Gung Memorial Hospital. The diagnosis of PD was based on the UK PD Society Brain Bank clinical diagnostic criteria by 2 neurologists specialized in movement disorders (YR Wu and CM Chen) [38]. Unrelated healthy adult volunteers matched for age, gender, ethnic origin, and area of residences were recruited as controls.

### Genetic Analysis

Genomic DNA was isolated from peripheral leukocytes by using a DNA Extraction Kit (Stratagene). *GRN* rs5848 polymorphism was determined using a pre-designed custom TaqMan SNP genotyping assay (assay ID: C7452046_20) on an ABI 7000 Real Time PCR system according to the manufacturer’s protocol (Applied Biosystems, Foster City, CA, USA). Briefly, each reaction included 20 ng of DNA, 0.9 µM of each primer, 0.2 µM of probe (probe sequence: TCTGCTCAGGCCTCCCTAGCACCTC[C/T]CCCTAACCAAATTCTCCCTGGACCC), and Universal PCR Master Mix (Applied Biosystems). PCR parameters were 50°C for 2 min, 95°C for 10 min, 40 cycles of 95°C for 15 sec, and 60°C for 1 min. The genotyping results were analyzed using SDS software version 1.1 (Applied Biosystems).

### Statistical Analysis

The genotypes of the patients and controls followed the Hardy–Weinberg equilibrium. Logistic regression analysis was carried out with PD as the dependent variable, and age, gender, and GRN genotypes as the independent variables. All *P* values were two-tailed. *P* values <0.025 were considered statistically significant to account for the multiple comparisons. Given the observed allele frequency in the present case-control study at a 0.025 significant level, we had power greater than 0.8 to identify an association when the per-allele genetic effect was greater than an odds ratio of 1.5 and 1.7 under a dominant and a recessive genetic model, respectively.

## References

[1] Lang AE, Lozano AM (1998) Parkinson’s disease. First of two parts. N Engl J Med 339: 1044–1053. 9761807 10.1056/NEJM199810083391506

[2] Dauer W, Przedborski S (2003) Parkinson’s disease: mechanisms and models. Neuron 39: 889–909. 12971891 10.1016/s0896-6273(03)00568-3

[3] Bhandari V, Bateman A (1992) Structure and chromosomal location of the human granulin gene. Biochem Biophys Res Commun 188: 57–63. 1417868 10.1016/0006-291x(92)92349-3

[4] Suzuki M, Lee HC, Kayasuga Y, Chiba S, Nedachi T, et al (2009) Roles of progranulin in sexual differentiation of the developing brain and adult neurogenesis. J Reprod Dev 55: 351–355. 19721334 10.1262/jrd.20249 PMC2739127

[5] He Z, Bateman A (1999) Progranulin gene expression regulates epithelial cell growth and promotes tumor growth in vivo. Cancer Res 59: 3222–3229. 10397269

[6] He Z, Ong CH, Halper J, Bateman A (2003) Progranulin is a mediator of the wound response. Nat Med 9: 225–229. 12524533 10.1038/nm816

[7] Rademakers R, Eriksen JL, Baker M, Robinson T, Ahmed Z, et al (2008) Common variation in the miR-659 binding-site of GRN is a major risk factor for TDP43-positive frontotemporal dementia. Hum Mol Genet 17: 3631–3642. 18723524 10.1093/hmg/ddn257 PMC2581433

[8] Jasinska-Myga B, Wider C, Opala G, Krygowska-Wajs A, Barcikowska M, et al (2009) GRN 3'UTR+78 C>T is not associated with risk for Parkinson’s disease. Eur J Neurol 16: 909–911. 19473366 10.1111/j.1468-1331.2009.02621.x

[9] Farrer MJ, Stone JT, Lin CH, Dachsel JC, Hulihan MM, et al (2007) Lrrk2 G2385R is an ancestral risk factor for Parkinson’s disease in Asia. Parkinsonism Relat Disord 13: 89–92. 17222580 10.1016/j.parkreldis.2006.12.001

[10] Ross OA, Wu YR, Lee MC, Funayama M, Chen ML, et al (2008) Analysis of Lrrk2 R1628P as a risk factor for Parkinson’s disease. Ann Neurol 64: 88–92. 18412265 10.1002/ana.21405

[11] Tan EK, Peng R, Teo YY, Tan LC, Angeles D, et al (2010) Multiple LRRK2 variants modulate risk of Parkinson disease: a Chinese multicenter study. Hum Mutat 31: 561–568. 20186690 10.1002/humu.21225

[12] Biskup S, Mueller JC, Sharma M, Lichtner P, Zimprich A, et al (2005) Common variants of LRRK2 are not associated with sporadic Parkinson’s disease. Ann Neurol 58: 905–908. 16254973 10.1002/ana.20664

[13] Paisan-Ruiz C, Evans EW, Jain S, Xiromerisiou G, Gibbs JR, et al (2006) Testing association between LRRK2 and Parkinson’s disease and investigating linkage disequilibrium. J Med Genet 43: e9. 16467219 10.1136/jmg.2005.036889 PMC2564648

[14] Satake W, Nakabayashi Y, Mizuta I, Hirota Y, Ito C, et al (2009) Genome-wide association study identifies common variants at four loci as genetic risk factors for Parkinson’s disease. Nat Genet 41: 1303–1307. 19915576 10.1038/ng.485

[15] Tan EK, Kwok HH, Tan LC, Zhao WT, Prakash KM, et al (2010) Analysis of GWAS-linked loci in Parkinson disease reaffirms PARK16 as a susceptibility locus. Neurology 75: 508–512. 20697102 10.1212/WNL.0b013e3181eccfcd PMC2918477

[16] Finch N, Baker M, Crook R, Swanson K, Kuntz K, et al (2009) Plasma progranulin levels predict progranulin mutation status in frontotemporal dementia patients and asymptomatic family members. Brain 132: 583–591. 19158106 10.1093/brain/awn352 PMC2664450

[17] Ghidoni R, Benussi L, Glionna M, Franzoni M, Binetti G (2008) Low plasma progranulin levels predict progranulin mutations in frontotemporal lobar degeneration. Neurology 71: 1235–1239. 18768919 10.1212/01.wnl.0000325058.10218.fc

[18] Sleegers K, Brouwers N, Van Damme P, Engelborghs S, Gijselinck I, et al (2009) Serum biomarker for progranulin-associated frontotemporal lobar degeneration. Ann Neurol 65: 603–609. 19288468 10.1002/ana.21621

[19] Van Damme P, Van Hoecke A, Lambrechts D, Vanacker P, Bogaert E, et al (2008) Progranulin functions as a neurotrophic factor to regulate neurite outgrowth and enhance neuronal survival. J Cell Biol 181: 37–41. 18378771 10.1083/jcb.200712039 PMC2287280

[20] Ahmed Z, Mackenzie IR, Hutton ML, Dickson DW (2007) Progranulin in frontotemporal lobar degeneration and neuroinflammation. J Neuroinflammation 4: 7. 17291356 10.1186/1742-2094-4-7 PMC1805428

[21] Hsiung GY, Fok A, Feldman HH, Rademakers R, Mackenzie IR (2011) rs5848 polymorphism and serum progranulin level. J Neurol Sci 300: 28–32. 21047645 10.1016/j.jns.2010.10.009 PMC3085023

[22] Baldereschi M, Di Carlo A, Rocca WA, Vanni P, Maggi S, et al (2000) Parkinson’s disease and parkinsonism in a longitudinal study: two-fold higher incidence in men. ILSA Working Group. Italian Longitudinal Study on Aging. Neurology 55: 1358–1363. 11087781 10.1212/wnl.55.9.1358

[23] Chen CC, Chen TF, Hwang YC, Wen YR, Chiu YH, et al (2009) Different prevalence rates of Parkinson’s disease in urban and rural areas: a population-based study in Taiwan. Neuroepidemiology 33: 350–357. 19887842 10.1159/000254572

[24] Rocca WA, Bower JH, Maraganore DM, Ahlskog JE, Grossardt BR, et al (2008) Increased risk of parkinsonism in women who underwent oophorectomy before menopause. Neurology 70: 200–209. 17761549 10.1212/01.wnl.0000280573.30975.6a

[25] Saunders-Pullman R (2003) Estrogens and Parkinson disease: neuroprotective, symptomatic, neither, or both? Endocrine 21: 81–87. 12777707 10.1385/ENDO:21:1:81

[26] Gillies GE, McArthur S (2010) Independent influences of sex steroids of systemic and central origin in a rat model of Parkinson’s disease: A contribution to sex-specific neuroprotection by estrogens. Horm Behav 57: 23–34. 19538962 10.1016/j.yhbeh.2009.06.002

[27] Simunovic F, Yi M, Wang Y, Stephens R, Sonntag KC (2010) Evidence for gender-specific transcriptional profiles of nigral dopamine neurons in Parkinson disease. PLoS One 5: e8856. 20111594 10.1371/journal.pone.0008856 PMC2810324

[28] Cantuti-Castelvetri I, Keller-McGandy C, Bouzou B, Asteris G, Clark TW, et al (2007) Effects of gender on nigral gene expression and parkinson disease. Neurobiol Dis 26: 606–614. 17412603 10.1016/j.nbd.2007.02.009 PMC2435483

[29] Spencer CC, Plagnol V, Strange A, Gardner M, Paisan-Ruiz C, et al (2011) Dissection of the genetics of Parkinson’s disease identifies an additional association 5' of SNCA and multiple associated haplotypes at 17q21. Hum Mol Genet 20: 345–353. 21044948 10.1093/hmg/ddq469 PMC3005904

[30] Saad M, Lesage S, Saint-Pierre A, Corvol JC, Zelenika D, et al (2011) Genome-wide association study confirms BST1 and suggests a locus on 12q24 as the risk loci for Parkinson’s disease in the European population. Hum Mol Genet 20: 615–627. 21084426 10.1093/hmg/ddq497

[31] Liu X, Cheng R, Verbitsky M, Kisselev S, Browne A, et al (2011) Genome-wide association study identifies candidate genes for Parkinson’s disease in an Ashkenazi Jewish population. BMC Med Genet 12: 104. 21812969 10.1186/1471-2350-12-104 PMC3166909

[32] Hamza TH, Zabetian CP, Tenesa A, Laederach A, Montimurro J, et al (2010) Common genetic variation in the HLA region is associated with late-onset sporadic Parkinson’s disease. Nat Genet 42: 781–785. 20711177 10.1038/ng.642 PMC2930111

[33] Edwards TL, Scott WK, Almonte C, Burt A, Powell EH, et al (2010) Genome-wide association study confirms SNPs in SNCA and the MAPT region as common risk factors for Parkinson disease. Ann Hum Genet 74: 97–109. 20070850 10.1111/j.1469-1809.2009.00560.x PMC2853717

[34] Simon-Sanchez J, Schulte C, Bras JM, Sharma M, Gibbs JR, et al (2009) Genome-wide association study reveals genetic risk underlying Parkinson’s disease. Nat Genet 41: 1308–1312. 19915575 10.1038/ng.487 PMC2787725

[35] Pankratz N, Wilk JB, Latourelle JC, DeStefano AL, Halter C, et al (2009) Genomewide association study for susceptibility genes contributing to familial Parkinson disease. Hum Genet 124: 593–605. 18985386 10.1007/s00439-008-0582-9 PMC2627511

[36] Fung HC, Scholz S, Matarin M, Simon-Sanchez J, Hernandez D, et al (2006) Genome-wide genotyping in Parkinson’s disease and neurologically normal controls: first stage analysis and public release of data. Lancet Neurol 5: 911–916. 17052657 10.1016/S1474-4422(06)70578-6

[37] Hamza TH, Chen H, Hill-Burns EM, Rhodes SL, Montimurro J, et al (2011) Genome-wide gene-environment study identifies glutamate receptor gene GRIN2A as a Parkinson’s disease modifier gene via interaction with coffee. PLoS Genet 7: e1002237. 21876681 10.1371/journal.pgen.1002237 PMC3158052

[38] Hughes AJ, Daniel SE, Kilford L, Lees AJ (1992) Accuracy of clinical diagnosis of idiopathic Parkinson’s disease: a clinico-pathological study of 100 cases. J Neurol Neurosurg Psychiatry 55: 181–184. 1564476 10.1136/jnnp.55.3.181 PMC1014720

